# A Novel Mutant of rLj-RGD3 (rLj-112) Suppressed the Proliferation and Metastasis of B16 Cells through the EGFR Signaling Pathway

**DOI:** 10.3390/md17020075

**Published:** 2019-01-23

**Authors:** Yuan-Yuan Zheng, Rong Xiao, Lu-Xin Zhang, Hui-Jie Yan, Ji-Hong Wang, Li Lv

**Affiliations:** 1 School of Life Sciences, Liaoning Normal University, Dalian 116081, China; bio_yy_zheng@163.com (Y.-Y.Z.); liulangmao1980@126.com (R.X.); zhangluxin_1992@163.com (L.-X.Z.); yhj_18342229453@163.com (H.-J.Y.); 2 Department of Pharmacology, Dalian Medical University, Dalian 116044, China

**Keywords:** rLj-RGD3, rLj-112, B16 cells, EGFR, internalization

## Abstract

Lj-RGD3, which contains three Arg–Gly–Asp (RGD) motifs, was first identified from the buccal glands of *Lampetra japonica* and has been shown to suppress the tumor progression in the previous studies. Apart from the three RGD motifs, Lj-RGD3 is also characterized by its high content of histidine in its amino acid sequence. In order to clarify whether the histidine-rich characterization of Lj-RGD3 is also associated with its anti-tumor activity, mutants were designed in which the three RGD motifs (Lj-112), or all histidines (Lj-27) or both (Lj-26) were deleted. Furthermore, a mutant (Lj-42) in which all histidines and three RGD motifs were respectively substituted with alanines and three Ala–Gly–Asp (AGD) motifs, as well as a mutant (Lj-41) in which all histidines were substituted with alanines was synthesized to avoid alterations in structure which might further cause changes in the peptides’ functions. After recombination and purification, recombinant Lj-112 (rLj-112), recombinant Lj-27 (rLj-27), recombinant Lj-41 (rLj-41), and recombinant Lj-RGD3 (rLj-RGD3) exhibited anti-proliferative activity in B16 cells, respectively; while recombinant Lj-26 (rLj-26) and recombinant Lj-42 (rLj-42) did not affect the proliferation of B16 cells significantly. In addition, the anti-proliferative activity of rLj-112 in B16 cells was due to apoptosis. Typical apoptosis features were observed, including chromatin condensation, fragmented DNA, and increased levels of cleaved caspase 3/caspase 7/nuclear enzyme poly (ADP-ribose) polymerase (PARP) in B16 cells. Similar to rLj-RGD3, rLj-112 was also capable of suppressing the migration and invasion of B16 cells by disturbing the F-actin arrangement. After labeling with FITC, rLj-112 was found localized in the cytoplasm of B16 cells, which induced the internalization of epidermal growth factor receptor (EGFR), suggesting that rLj-112 might block the EGFR mediated signaling pathway. Actually, the phosphorylation level of EGFR and its downstream signal molecules including Akt, PI3K, p38, and ERK1/2 was reduced in the rLj-112 treated B16 cells. In vivo, rLj-112 also inhibited the growth, weight, and volume of the tumors in B16 xenografted C57BL/6 mice without reducing their body weight, indicating that rLj-112 might be safe and might be used as an effective anti-tumor drug in the near future.

## 1. Introduction

Histidine-rich glycoprotein (HRG) is an α2-plasma glycoprotein which was first isolated from human serum in 1972 [1,2]. Usually, HRG is synthesized by liver parenchymal cells and is maintained at relatively high concentrations in plasma [3]. As a result of the properties of binding to a variety of ligands including heparin, heparan sulphate, plasminogen, fibrinogen, IgG, FcγR, and C1q, HRG has been reported to participate in the regulation of thrombosis, angiogenesis, immunity, cell adhesion, proliferation, and migration processes [4,5,6,7,8]. In 2011, Rolny and colleagues reported that the expression level of HRG in tumor tissues was weaker than that found in healthy counterparts [9]. Very recently, HRG was proved to suppress tumor growth and metastasis by inducing macrophage polarization and vessel normalization, as well as regulating platelet activity [9,10,11]. Thus, HRG might have prognostic value and its synthetic peptides might be used in the treatment of cancers [4,9,12].

Lj-RGD3, which contains three Arg–Gly–Asp (RGD) motifs, was firstly identified from the buccal glands of the hematophagous *Lampetra japonica* (*L. japonica*) [13]. In previous studies, recombinant Lj-RGD3 (rLj-RGD3) has been demonstrated to inhibit platelet aggregation, angiogenesis, tumor growth, and metastasis in an integrin-dependent pathway [13,14,15,16,17,18]. Furthermore, rLj-RGD3 was also found to exhibit antifungal activity against *Candida albicans* (*C. albicans*) [19]. This suggests that Lj-RGD3 is a very important protein in the buccal glands of *L. japonica*, which would help these bloodsuckers (*L. japonica*) suppress the side effects generated from the host fishes. Apart from the three RGD motifs, Lj-RGD3 is also characterized by its high content of histidine in its amino acid sequence. Sequence alignment revealed that Lj-RGD3 shares 40% identity with a HRG (from amino acid residue 21 to residue 102) from *Brugia malayi* (*B. malayi*), 41% identity with a T-cell receptor β chain ANA 11 (from residue 5 to residue 93) from *B. malayi*, 30% identity with theHis/Pro-rich domain (from residue 330 to residue 389) of the human HRG, and 36% identity with a His/Gly-rich domain (from residue 408 to residue 468) of the human high-molecular-weight kininogen (HK) [19]. Since HRG treatment could also reduce the growth and metastasis of tumor cells [10], the anti-tumor mechanism of Lj-RGD3 might not only depend on the interactions between the three RGD motifs and integrins. In other words, when Lj-RGD3 lacks the three RGD motifs, it might also exhibit the anti-tumor property due to its histidine-rich characterization. With this in mind, mutants which deleted the three RGD motifs (Lj-112), or all histidines (Lj-27), or both (Lj-26) were obtained in the present study. Furthermore, a mutant (Lj-42) in which all histidines and three RGD motifs were respectively substituted with alanines and three Ala–Gly–Asp (AGD) motifs, as well as a mutant (Lj-41) in which all histidines were substituted with alanines was synthesized to avoid alterations in structure which might further cause changes in the peptides’ functions. In addition, the anti-tumor effects and mechanisms of rLj-112 on B16 melanoma were assessed from both in vitro and in vivo studies.

## 2. Results

### 2.1. rLj-112 Could Significantly Inhibit B16 Cells’ Proliferation by Inducing Apoptosis

**Figure 1:**
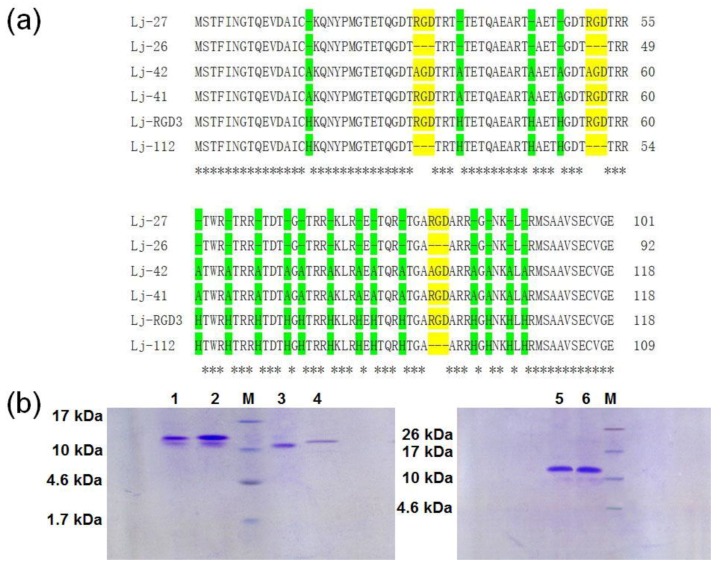
Lj-RGD3 and its mutants. (**a**) The amino acid sequences of Lj-27, Lj-26, Lj-42, Lj-41, Lj-RGD3, and Lj-112. RGD or AGD motifs are indicated with yellow; histidines or alanines are indicated with green. Dashes (-) indicate gaps inserted into the alignment. Asterisks (*) indicate the identical residues. (**b**) The purified rLj-RGD3, rLj-112, rLj-27, rLj-26, rLj-41, and rLj-42 were detected by 16.5% Tricine SDS-PAGE. M, low molecular weight protein marker; lane 1, rLj-112; lane 2, rLj-RGD3; lane 3, rLj-26; lane 4, rLj-27; lane 5, rLj-41; lane 6, rLj-42.

**Figure 2:**
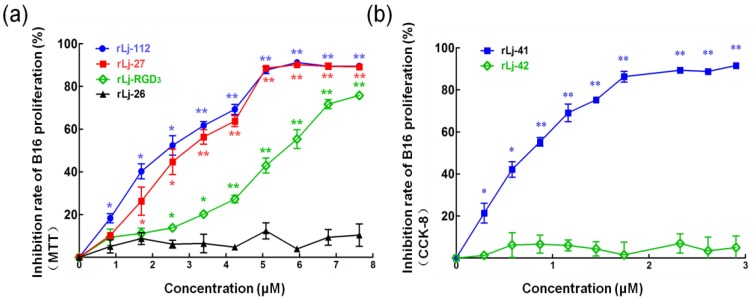
rLj-RGD3 and its mutants suppressed the proliferation of B16 cells in a dose-dependent manner. (**a**) The B16 cells were treated with the same concentrations (0, 0.85, 1.70, 2.55, 3.40, 4.25, 5.10, 5.95, 6.8, and 7.65 μM) of rLj-RGD3, rLj-112, rLj-27 and rLj-26 at 37 °C for 24 h. MTT assays were used to measure the inhibitory rates of rLj-RGD3, rLj-112, rLj-27, and rLj-26 on the proliferation of B16 cells. (**b**) The effects of rLj-41 and rLj-42 on the proliferation of B16 cells were assayed by CCK-8. The significant differences of inhibitory rates between the control and rLj-RGD3/rLj-112/rLj-27/rLj-26/rLj-41/rLj-42 treated groups are indicated with asterisks (*: *p* < 0.05; **: *p* < 0.01).

With the exception of Lj-RGD3, Lj-112, Lj-27, Lj-26, Lj-41, and Lj-42 were synthesized based on the sequences listed in Figure 1a. According to the previous studies, rLj-RGD3, recombinant Lj-112 (rLj-112), recombinant Lj-27 (rLj-27), recombinant Lj-26 (rLj-26), recombinant Lj-41 (rLj-41), and recombinant Lj-42 (rLj-42) were respectively expressed as the soluble proteins in *Escherichia coli* BL21 cells [19]. After purification through a His-tag affinity column, rLj-RGD3, rLj-112, rLj-27, rLj-26, rLj-41, and rLj-42 could be detected mainly as a single band on Tricine sodium dodecyl sulfate-polyacrylamide gel electrophoresis (SDS-PAGE) (Figure 1b). In addition, the molecular weights of rLj-RGD3, rLj-112, rLj-27, rLj-26, rLj-41, and rLj-42 are about 14.5 kDa, 13.5 kDa, 12.1 kDa, 11.1 kDa, 13.3 kDa and 13.1 kDa, respectively [19]. In order to further clarify whether the mutants of rLj-RGD3 still possess the anti-tumor activity, 3-(4,5-dimethylthiazol-2-yl)-2, 5-diphenyltetrazolium bromide (MTT) and cell counting kit-8 (CCK-8) assays were performed. As Figure 2 shows, rLj-RGD3, rLj-112, and rLj-27 were able to reduce the proliferation of B16 cells in a dose-dependent manner. Furthermore, the IC_50_ values for rLj-RGD3, rLj-112, and rLj-27 were 5.72 μM, 2.53 μM and 3.01 μM, respectively. Similar to rLj-27, rLj-41 was also able to inhibit the proliferation of B16 cells dose-dependently as it contains three RGD motifs (Figure 2). However, rLj-26 did not show any inhibitory effects on the proliferation of B16 cells. In accordance with the results of rLj-26, rLj-42 did not inhibit the proliferation of B16 cells as the three RGD motifs and histidines in its amino acid sequence were substituted with three AGD motifs and alanines, respectively (Figure 2). In order to illuminate whether the histidine-rich characterization of rLj-RGD3 is associated with its anti-tumor activity, rLj-112 was chosen for the following experiments.

**Figure 3:**
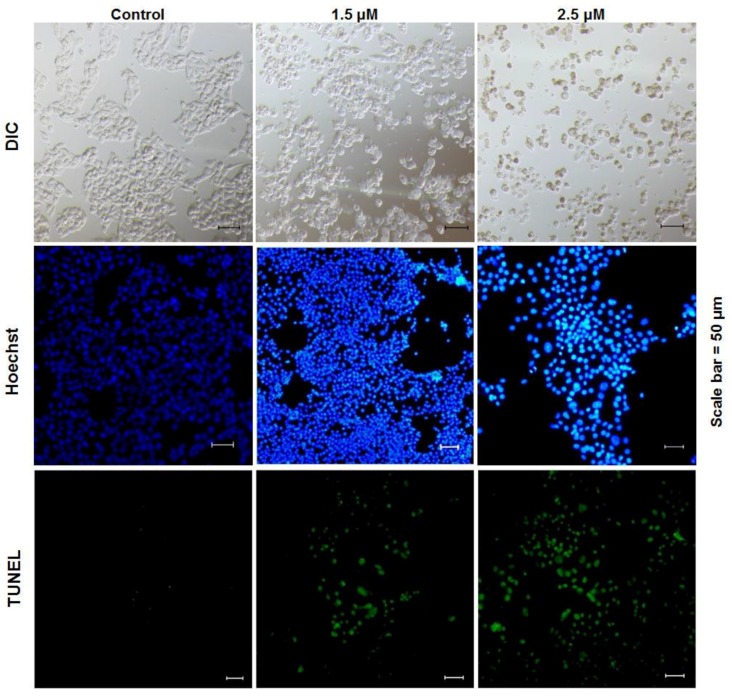
rLj-112 induced apoptosis in B16 cells. The B16 cells were pretreated with PBS, 1.5 and 2.5 μM rLj-112 and then stained with Hoechst 33258 and a TUNEL Apoptosis Detection Kit. The shape of the B16 cells is shown in differential interference contrast (DIC) mode (the first line). The blue signals indicate the nuclei in the B16 cells stained with Hoechst 33258 (the second line); while the green signals indicate the apoptotic B16 cells (the third line). Scale bar = 50 μm.

**Figure 4:**
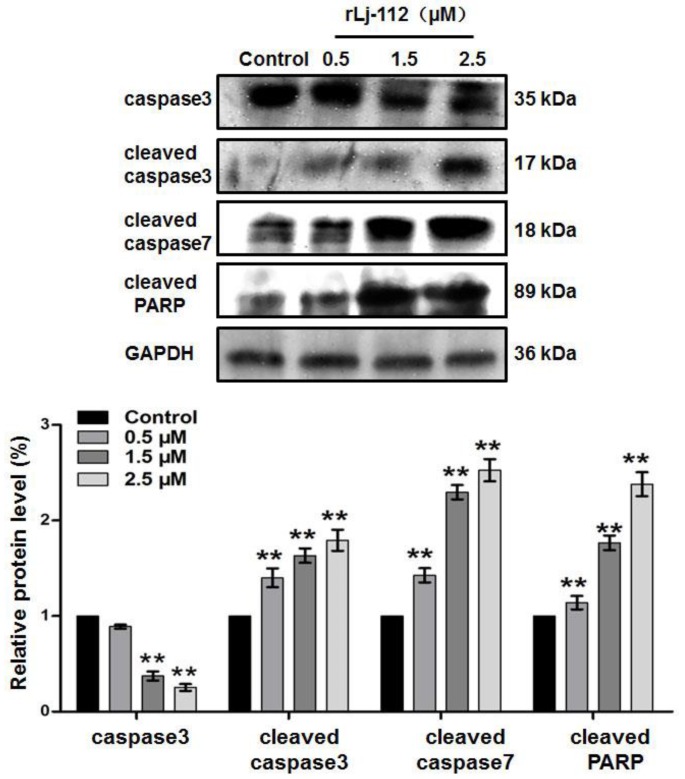
rLj-112 changed the protein levels of caspase 3, cleaved caspase 3, cleaved caspase 7, and cleaved PARP in B16 cells. Western blot showed the caspase 3, cleaved caspase 3, cleaved caspase 7, and cleaved PARP in B16 cells treated with PBS, 0.5, 1.5, and 2.5 μM rLj-112. Glyceraldehyde-3-phosphate dehydrogenase (GAPDH) was used as a loading control. A histogram shows the intensity of caspase 3, cleaved caspase 3, cleaved caspase 7, and cleaved PARP in the B16 cells in the presence of PBS and rLj-112. The significant differences between the control and rLj-112 treated groups are indicated with asterisks (**: *p* < 0.01). The full images of the western blot data are shown in Figure S1.

According to observations using a confocal microscope, the B16 cells lost their original shape in the presence of rLj-112 (Figure 3). In the phosphate buffered saline (PBS) group (control group), the shape of the B16 cells was spindlelike. After treatment with rLj-112, the B16 cells gradually became round and shrank (Figure 3). To examine whether the anti-proliferative effects of rLj-112 on B16 cells were due to apoptosis, the PBS or rLj-112 treated B16 cells were stained with Hoechst 33258 and a TdT-UTP nick end labeling (TUNEL) Apoptosis Detection Kit, respectively (Figure 3). The Hoechst 33258 staining assay showed the nuclei in the rLj-112 treated cells were brighter than those in the PBS treated B16 cells. The rLj-112 treated B16 cells also had the typical characterizations of apoptotic cells, such as chromatin shrinking. While the TUNEL staining assay showed the proportion of FITC-labeled B16 cells increased in the rLj-112 treated groups when compared with the control cells, indicating that rLj-112 could trigger DNA damage in B16 cells (Figure 3). As we all know, caspase 3 and caspase 7 are executive proteins during apoptosis, which could cause cleavage of key proteins downstream like the nuclear enzyme poly (ADP-ribose) polymerase (PARP) [20]. On the basis of the analysis of our western blot, rLj-112 was able to up-regulate the level of cleaved caspase 3, cleaved caspase 7, and cleaved PARP in the B16 cells; while the level of caspase 3 in the B16 cells was reduced in the presence of rLj-112 (Figure 4). The above results indicate that the anti-proliferative effects of rLj-112 on B16 cells were due to apoptosis, which is similar to those of rLj-RGD3.

### 2.2. rLj-112 Inhibited Metastasis of B16 Cells by Disrupting Cytoskeleton in B16 Cells

**Figure 5:**
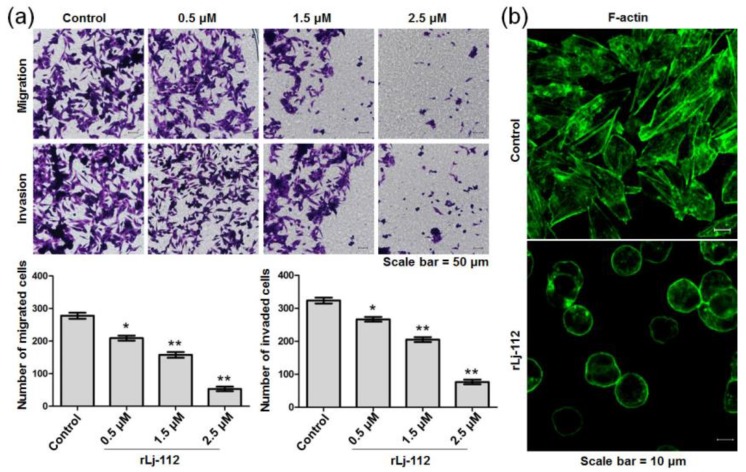
rLj-112 inhibited the migration and invasion of B16 cells by disturbing the F-actin arrangement. (**a**) After treatment with PBS, 0.5, 1.5, and 2.5 μM rLj-112, an inverted microscope was used to observe the migrated and invaded B16 cells. Scale bar = 50 μm. The histograms show the number of the migrated and invaded B16 cells in the presence of PBS and rLj-112. The significant differences between the control and rLj-112 treated groups are indicated with asterisks (*: *p* < 0.05; **: *p* < 0.01). (**b**) After treatment with 0 or 2.5 μM rLj-112, F-actin (green signals) in the B16 cells was visualized by staining with FITC-labeled phalloidine. Scale bar = 10 μm.

The migration and invasion of melanoma cells are closely associated with the malignant metastasis of melanomas. To determine whether rLj-112 was able to inhibit metastasis of B16 melanoma cells, a transwell system was chosen in the present study. Compared with the control group, rLj-112 was found to inhibit the migration of B16 cells significantly. rLj-112 (0.5, 1.5, and 2.5 μM) suppressed the migration of B16 cells to 75.2% (*p* < 0.05), 56.8% (*p* < 0.01), and 19.1% (*p* < 0.01) of the control (Figure 5a). A similar situation also occurred in the invasion assay. Compared with the control group, rLj-112 was also observed to inhibit the invasion of B16 cells (Figure 5a). Furthermore, the inhibitory rate of the B16 cells’ invasion in the presence of 0.5, 1.5, and 2.5 μM of rLj-112 was 17.3% (*p* < 0.05), 36.5% (*p* < 0.01), and 76.2% (*p* < 0.01), respectively. Cytoskeleton arrangement is one of the most important factors associated with the migration and invasion processes of tumor cells [21]. Thus, we also analyzed the cytoskeleton arrangement in the B16 cells in the presence of rLj-112 using FITC-labeled phalloidine. In contrast with the typical F-actin arrangement in the control group, rLj-112 significantly destroyed the F-actin arrangement in the B16 cells (Figure 5b). After treatment with rLj-112 (2.5 μM), the architecture of F-actin in the B16 cells became discontinuous.

### 2.3. rLj-112 Suppressed the Activation of Epidermal Growth Factor Receptor (EGFR) Pathway in B16 Cells

**Figure 6:**
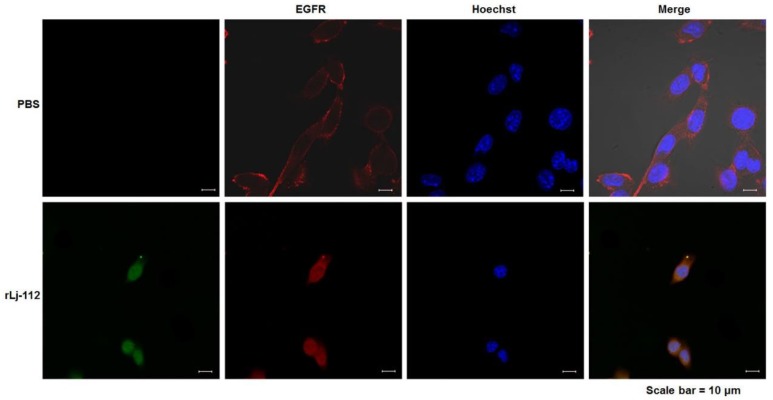
rLj-112 induced the internalization of EGFR in B16 cells. The PBS treated B16 cells were used as the control group. The localization of FITC-labeled rLj-112 in the B16 cells is shown in green. The localization of EGFR and nuclei in the PBS and FITC-labeled rLj-112 treated B16 cells is shown in red and blue, respectively. Scale bar = 10 μm.

**Figure 7:**
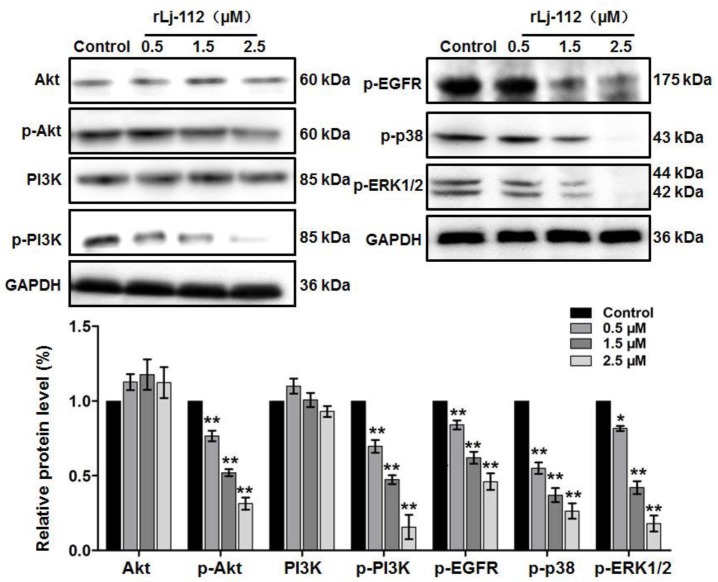
rLj-112 suppressed the activation of EGFR pathway in B16 cells. Western blots show the levels of Akt, p-Akt, PI3K, p-PI3K, p-EGFR, p-p38, and p-ERK1/2 in B16 cells treated with PBS, 0.5, 1.5 and 2.5 μM rLj-112, respectively. GAPDH served as a loading control. A histogram indicates the intensity of the Akt, p-Akt, PI3K, p-PI3K, p-EGFR, p-p38, and p-ERK1/2 in the B16 cells. The significant differences between the control and rLj-112 treated groups are indicated with asterisks (*: *p* < 0.05; **: *p* < 0.01). The full images of the western blot data are shown in Figure S2.

Previous studies have shown that the EGFR pathway plays an important role in the proliferation, migration, and invasion of cancer cells [22,23]. Thus, we also analyzed the effects of rLj-112 on the EGFR pathway. As shown in Figure 6, EGFR (red) showed a membrane staining pattern in the PBS treated B16 cells; however, after treating with FITC-labeled rLj-112 (green), both green and red signals were localized within the cytoplasm of the B16 cells. Furthermore, our western blot analysis showed that rLj-112 treatment could decrease the levels of p-EGFR, p-protein kinase B (Akt), p-phosphoinositide 3-kinase (PI3K), p-p38, and p-extracellular signal-regulated kinase (ERK)1/2 in a dose-dependent manner in B16 cells (Figure 7).

### 2.4. rLj-112 Inhibited Tumor Growth in the B16 Xenografted Model

**Figure 8:**
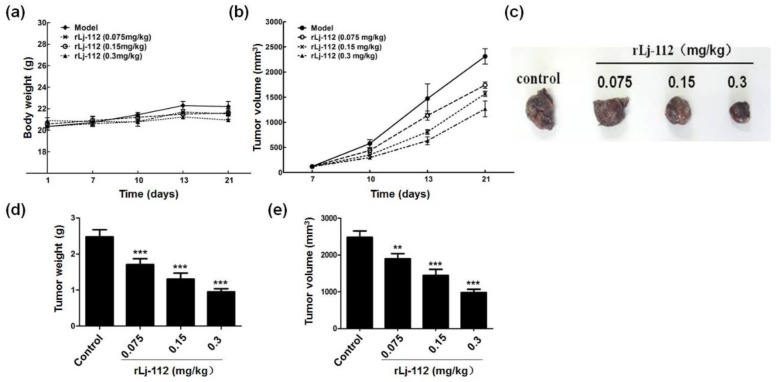
rLj-112 inhibited the tumor growth in C57BL/6 mice xenografted with B16 cells. (**a**,**b**) The xenografted C57BL/6 mice were treated with normal saline or different concentrations of rLj-112 for 21 days, and their body weights and tumor volumes were recorded. (**c**) After 21 days, these C57BL/6 mice were sacrificed and the xenografted tumors were isolated and captured. (**d**,**e**) The weight and volume of the xenografted tumors. The significant differences between the normal saline and rLj-112 treated groups are indicated with asterisks (**: *p* < 0.01; ***: *p* < 0.001).

Since rLj-112 was capable of suppressing the growth and metastasis of B16 cells in vitro studies, the anti-tumor activity of rLj-112 was also evaluated in mouse melanoma (B16) xenografted models. As shown in Figure 8a, 0.075, 0.15, and 0.3 mg/kg rLj-112 did not reduce the body weight of the tumor-bearing mice when compared with the mice administered with normal saline. Effectively, rLj-112 at different concentrations decreased the tumor growth significantly in B16 xenografted models (Figure 8b). After 21 days, rLj-112 was shown to reduce the weight and volume of the isolated tumors significantly in a dose-dependent manner (Figure 8c–e). The tumor weights were 2.48 ± 0.20 g, 1.93 ± 0.16 g (*p* < 0.001), 1.38 ± 0.20 g (*p* < 0.001), and 0.96 ± 0.08 g (*p* < 0.001) in the normal saline group, and 0.075, 0.15, and 0.3 mg/kg rLj-112 treated groups, respectively (Figure 8d). In addition, the tumor volumes were 2480.3 ± 174.4 mm^3^, 1896.8 ± 143.0 mm^3^ (*p* < 0.01), 1442.5 ± 170.0 mm^3^ (*p* < 0.001), and 978.8 ± 90.4 mm^3^ (*p* < 0.001) in the presence of normal saline, 0.075, 0.15, and 0.3 mg/kg rLj-112, respectively (Figure 8e). Furthermore, inhibitory rate values for tumor volumes in the 0.075, 0.15, and 0.3 mg/kg rLj-112 treated groups were 23.5% (*p* < 0.01), 41.84% (*p* < 0.001), and 60.53% (*p* < 0.001), respectively.

## 3. Discussion

In previous studies, rLj-RGD3 was shown to inhibit the growth and metastasis of endothelial cells (ECV 304 cells) and tumor cells including breast carcinoma drug-resistant MCF-7/Adr cells, drug-sensitive MCF-7 cells, ovarian carcinoma HeyA8 cells, renal carcinoma 786-0 cells, and pancreatic carcinoma Panc-1 cells [13,14,15,16,17,18]. Wang and colleagues attributed the anti-tumor activities of rLj-RGD3 to its integrin binding properties [13]. Aside from the three RGD motifs, rLj-RGD3 is also characterized as a histidine-rich protein which contains 17 histidines in its amino acid sequence [19]. At present, HRG is known as an important protein in the suppression of tumor progression [9,12,24]. Therefore, we speculated that rLj-RGD3 might have two functional motifs to suppress the tumor growth and metastasis. One is the three RGD motifs which could bind specific integrins to trigger apoptosis in tumor cells; the other is the histidine-rich motif which would recognize other targets in tumor cells to induce apoptosis. In the present study, the mutant rLj-112 in which the three RGD motifs were removed, the mutant rLj-27 in which all the histidines were removed, and the mutant rLj-41 in which all histidines were substituted with alanines were shown to inhibit the proliferation of B16 cells; while mutant rLj-26 which lacks both the three RGD motifs and histidine residues, as well as mutant rLj-42 in which all histidines and RGD motifs were respectively substituted with alanines and AGD motifs did not show any inhibitory effects on the proliferation of B16 cells. This confirmed that the three RGD motifs and histidine-rich motif are responsible for the anti-tumor activities of rLj-RGD3. Very interestingly, either rLj-112 or rLj-27 at the same dosages showed better inhibitory effects on the proliferation of B16 cells than rLj-RGD3. This is similar to the study reported by Wu and colleagues, in which the antifungal activity of rLj-112 was stronger than that of rLj-RGD3 [19]. The authors speculated that missing either the three RGD motifs or the histidine-rich motif would lead to the change in the spatial structure of rLj-RGD3, which finally resulted in the different activities of rLj-RGD3 and its mutants (rLj-112 and rLj-27). In other words, rLj-112 and rLj-27 might be liable to bind to their targets to initiate the anti-tumor steps. However, the detailed mechanism still needs further study.

According to our previous studies, the purity of rLj-RGD3 was 99.94% through high-performance liquid chromatography (HPLC) analysis [15]. Furthermore, the purified rLj-RGD3 migrated as two bands on Tricine SDS-PAGE. After adding vitamin C, a strong reducing agent, the purified rLj-RGD3 would migrate as a single band on the Tricine SDS-PAGE [15]. The incomplete reduction of the disulfide bonds that remained in the purified rLj-RGD3 and rLj-112 might lead to the two bands on Tricine SDS-PAGE.

Triggering DNA damage has been identified as an effective strategy in cancer therapy [25]. Similar to rLj-RGD3, rLj-112 could also inhibit the proliferation of B16 cells by inducing apoptosis as typical apoptosis features including chromatin condensation (Hoechst 33258 staining) and fragmented DNA (TUNEL assay) were very evident in the rLj-112 treated B16 cells. Additionally, apoptosis in the B16 cells induced by rLj-112 was initiated via a caspase-dependent pathway. This is coincident with the apoptotic mechanisms induced by rLj-RGD3 [15]. Although rLj-112 lacks the three RGD motifs, it still possesses the abilities to inhibit the migration and invasion of B16 cells by disturbing the arrangement of F-actin in B16 cells. With the above integration, rLj-112 was intraperitoneally injected into C57BL/6 mice xenografted with B16 cells and was able to effectively suppress the tumor growth in those C57BL/6 mice based on our in vivo studies. Thus, rLj-112 might be used as an effective anti-tumor drug in the future studies.

EGFR is a very important transmembrane receptor which belongs to the receptor tyrosine kinases (RTKs) superfamily [23,26,27]. A large number of studies have reported that EGFR could activate intracellular signaling cascades and initiate the proliferation, differentiation, migration, and survival of cells [23,28]. Recently, EGFR has been used as an anti-tumor target due to its relationship with tumor progression [23,27,29,30]. On the basis of the observation of confocal microscope, EGFR usually showed a membrane staining pattern in the PBS treated B16 cells, while rLj-112 could induce the internalization of EGFR, suggesting that rLj-112 might affect EGFR pathway. Interestingly, both rLj-112 and EGFR were found internalized in the cytoplasm of B16 cells. As early as 1999–2001, Xu and Gyurko reported that histatin 3 and histatin 5, which are histidine-rich proteins from human saliva, could be internalized in *C. albicans* only after a period of binding to their surface [31,32]. In addition, the authors held that the internalization of histatin 3 and histatin 5 is closely associated with cell death [31,32]. As rLj-112 contains a high content of histidine residues in its amino acid sequence, this characterization might lead to its internalization in the cytoplasm of B16 cells and subsequently cause the death of B16 cells. In addition, previous studies have also shown that EGFR and its ligand could be internalized via clathrin coated pits [27]. Furthermore, the internalized EGFR could be either recycled to the membrane or degraded in the lysosome [27]. However, whether the internalization of both rLj-112 and EGFR is due to the fate of EGFR still needs further investigation.

**Figure 9:**
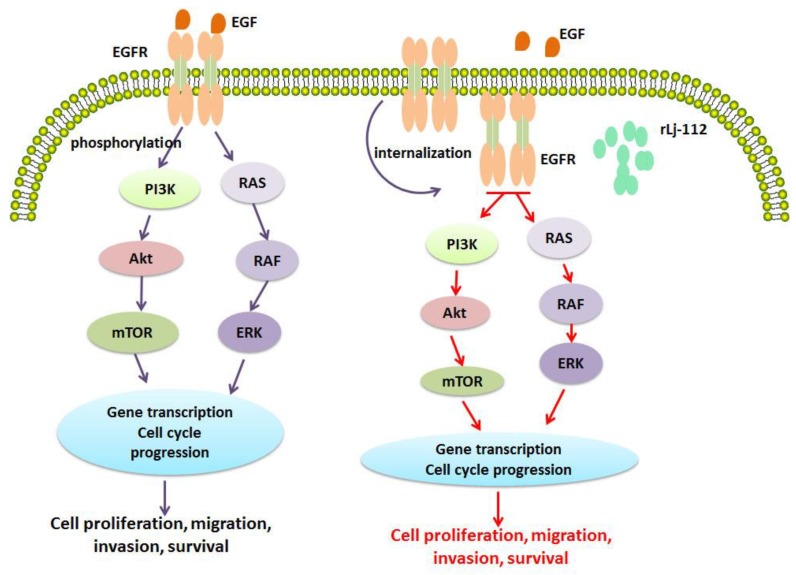
rLj-112 was internalized with EGFR to suppress the downstream signaling pathways to initiate the anti-tumor steps in B16 cells. Epidermal growth factor (EGF) or other ligands might target EGFR (labeled with orange) on the membrane of the B16 cells to activate the downstream signaling pathways including PI3K, Akt, and MAPK (increased phosphorylation levels) to promote cell proliferation, migration, invasion, and survival. After treatment with FITC-labeled rLj-112 (green), both rLj-112 and EGFR (orange) were internalized into the cytoplasm of the B16 cells and subsequently the downstream signaling pathways including PI3K, Akt, and MAPK were inhibited (decreased phosphorylation levels) which finally led to the suppression of cell proliferation, migration, invasion, and survival. Promotion is indicated with purple arrows; while suppression is indicated with red arrows.

Previous studies have reported that some EGFR inhibitors such as cetuximab could bind to EGFR, which would result in the blockage of its downstream signaling pathway via EGFR internalization [33,34,35,36]. This is consistent with our observation as the phosphorylation level of EGFR, and its downstream signal molecules including Akt, PI3K, p38, and ERK1/2, was reduced in the rLj-112 treated B16 cells. This indicated that rLj-112 might be able to suppress the activation of the EGFR signaling pathway effectively. Since both PI3K/Akt and mitogen-activated protein kinase (MAPK) signaling pathways are involved in the modulation of cell survival, proliferation, migration, and invasion [37,38,39], the anti-tumor activity of rLj-112 might be attributed to its inhibition on the EGFR and its downstream PI3K/Akt and MAPK signaling pathways (Figure 9).

## 4. Materials and Methods

### 4.1. Preparation of rLj-RGD3 and Its Mutants

In the aforementioned study, the mutant of Lj-RGD3 without the three RGD motifs was named Lj-112; while the mutant of Lj-RGD3 without the three RGD motifs and all histidines was named Lj-26 [19]. The rLj-RGD3, rLj-112, and rLj-26 were obtained based on the method reported by Wang and Wu [13,19]. In the present study, the Lj-RGD3 mutant without all histidines was named Lj-27; the mutant in which all histidines and RGD motifs were respectively substituted with alanines and AGD motifs was named Lj-42; and the mutant in which all histidines were substituted with alanines was named rLj-41. Subsequently, the synthesized Lj-27, Lj-41, and Lj-42 were subcloned into a pET23b vector (TaKaRa Company, Dalian, China). After 1 mM isopropyl-1-thio-β-D-galactopyranoside (IPTG) induction, rLj-27, rLj-41, and rLj-42 were purified through a His-Bind column and analyzed by 16.5% Tricine SDS-PAGE. The concentration of rLj-RGD3, rLj-112, rLj-27, rLj-26, rLj-41, and rLj-42 was measured by a bicinchoninic acid (BCA) kit based on the standard curve of bovine serum albumin (BSA).

### 4.2. Cell Culture

B16 cells were purchased from the Cell Bank of Chinese Academy of Sciences and were grown in RPMI 1640 medium (Thermo Fisher Scientific, Waltham, MA, USA) supplied with 10% fetal bovine serum (FBS, Thermo Fisher Scientific, Waltham, MA, USA), penicillin (100 IU/mL), and streptomycin (100 mg/mL, Gibco, Grand Island, NY, USA) at 37 °C in a thermostatic incubator (5% CO_2_).

### 4.3. Proliferation Assay

MTT assay was used to analyze the effects of rLj-RGD3, rLj-112, rLj-26, and rLj-27 on B16 cells’ proliferation. B16 cells (5 × 10^3^ cells/well) were seeded on 96-well plates. After treatment with different concentrations of rLj-RGD3, rLj-112, rLj-26, or rLj-27 for 24 h, MTT solution (10 mg/mL, final concentration) was added into each well. Four hours later, the supernatant was removed and dimethyl sulfoxide (DMSO, 100 μL) was added into the wells. The absorbance at 570 nm of the above cells was determined using a microplate reader (Bio-Rad Inc., Hercules, CA, USA). All the experiments were repeated three times. Furthermore, the effects of rLj-41 and rLj-42 on the proliferation of B16 cells were also analyzed by CCK-8 (Meilun Biotechnology, Dalian, China). Similarly, the B16 cells were also treated with rLj-41 and rLj-42 (0, 0.216, 0.432, 0.648, 0.864, 1.08, 1.29, 1.72, 1.94, and 2.16 μM) for 24 h. Subsequently, CCK-8 solution was added into the above cells and incubated at 37 °C for 4 h. Absorbance at 492 nm of the above cells was determined with the microplate reader.

### 4.4. Fluorescent Staining

Firstly, B16 cells were cultured on the slides placed in the 6-wells. And then the cells were treatment with PBS or different concentrations of rLj-112 (1.5 and 2.5 μM) for 24 h. Subsequently, the cells were fixed with 4% paraformaldehyde for 1 h. In the Hoechst 33258 staining assay, the above cells were stained with Hoechst 33258 (Beyotime Biotechnology, Shanghai, China) for 15 min in the dark; in the TUNEL assay, the above cells were permeabilized with 0.1% Triton X-100 for 2 min and then stained with TUNEL Apoptosis Detection Kit (Beyotime Biotechnology, Shanghai, China) according to the manufacturer’s instructions; in the cytoskeleton detection, FITC-labeled phalloidine was used to recognize the F-actin in B16 cells. Finally, the stained cells were observed by a laser scanning confocal microscopy (Carl Zeiss, Freiburg, Germany).

### 4.5. Western Blotting

B16 cells seeded in the 100-mm-diameter plates (Thermo Scientific, USA) were treated with PBS or rLj-112 at different concentrations (0.5, 1.5, and 2.5 μM) for 24 h at 37 °C. The cells were lysed with lysis buffer for 15 min at 4 °C and the supernatant was harvested after centrifugation. The cell lysates were subsequently resolved on SDS-PAGE and then transferred onto the nitrocellulose membranes (Millipore, Darmstadt, Germany), respectively. After blocking, the primary antibodies were incubated with the above membranes, respectively. The dilution ratios for the primary antibodies used in the present study are listed as followed: caspase 3 antibody (Proteintech, Rosemont, IL, USA; 1:1000); caspase 7 antibody (Cell Signalling Technology, Danvers, MA, USA; 1:1000); PARP antibody (Cell Signalling Technology, Danvers, MA, USA; 1:1000); Akt antibody (Cell Signalling Technology, Danvers, MA, USA; 1:1000); phospho-Akt (Ser473) antibody (Cell Signalling Technology, Danvers, MA, USA; 1:1000); PI3K antibody (Cell Signalling Technology, Danvers, MA, USA; 1:1000); phospho-PI3K (Tyr458 + Tyr199) antibody (Cell Signalling Technology, Danvers, MA, USA; 1:1000); phospho-EGFR antibody (Cell Signalling Technology, Danvers, MA, USA; 1:1000); phospho-p38 (Thr180 + Tyr182) antibody (Cell Signalling Technology, Danvers, MA, USA; 1:1000); phospho-ERK1/2 (Thr202 + Tyr204) antibody (Cell Signalling Technology, Danvers, MA, USA; 1:1000), and GAPDH antibody (Cell Signalling Technology, Danvers, MA, USA; 1:1000). After washing with PBS five times, the membranes were also incubated with horseradish peroxidase (HRP)-conjugated secondary antibodies (Thermo Fisher Scientific, Waltham, MA, USA). Finally, enhanced chemiluminescence (Santa Cruz, Dallas, TX, USA) was used to detect the signals. All experiments were performed in triplicate. The bands were scanned using an image scanning densitometer (Proteinsimple, San Jose, CA, USA).

### 4.6. Migration and Invasion Assays

Migration and invasion assays were respectively performed using a transwell system (8.0 mm pore size, Corning, Corning, NY, USA). Briefly, B16 cells (1 × 10^5^ cells) in 200 μL FBS-free medium were incubated with 0.5, 1.5, and 2.5 μM rLj-112 for 30 min and then were added into the upper chamber, respectively. Next, the 1640 medium with FBS and basic fibroblast growth factor (bFGF, 3 ng/mL, final concentration) was added into the lower chamber. The different groups were incubated in the same conditions at 37 °C for 16 h in the migration assay and for 24 h in the invasion assay, respectively. Furthermore, the matrigel basement membrane matrix (4.0 mg/mL, final concentration, 200 μL, BD, Franklin Lakes, NJ, USA) was precoated on the upper surface of the cellulose acetate membrane in the transwell, and then the PBS or rLj-112 treated B16 cells were added into the transwell in the invasion assay. Non-migrant and non-invasive cells were removed from the upper surface of the cellulose acetate membrane by washing with PBS. Subsequently, the migrated and invaded B16 cells were fixed with 4% paraformaldehyde and stained with Giemsa solution for 7 min. After washing with H_2_O, the migrated and invaded B16 cells were imaged with an optical microscope (Nikon, Tokyo, Japan). In addition, the number of migrated and invaded B16 cells was summarized by a soft ware named as Dot counter.

### 4.7. Immunofluorescent Staining

Firstly, rLj-112 was conjugated with FITC by using a HOOK™ FITC Labeling Kit (G-Bioscience, Louis, MO, USA) according to the manufacture’s instructions. Secondly, the B16 cells on the climbing films were respectively treated with PBS and 2.5 μM FITC-labeled rLj-112 for 7 min. Thirdly, these cells were fixed with 4% paraformaldehyde for 30 min at room temperature, respectively. After washing with PBS three times, the slides were blocked with goat serum (Solarbio, Beijing, China) for 40 min and incubated with a rabbit anti-EGFR antibody (Cell Signalling Technology, Danvers, MA, USA; 1: 50) at 37 °C for 1 h. After washing with PBS three times, the slides were incubated with AlexaFluor 594 conjugated Donkey Anti-Rabbit IgG (Proteintech, Rosemont, IL, USA) for 30 min at 37 °C. Finally, the above cells were stained with Hoechst 33258 (Beyotime Biotechnology, Shanghai, China) for 5 min in the dark. The images were captured using the laser scanning confocal microscopy.

### 4.8. Xenograft Models

Female C57BL/6 mice aged at 6 weeks were purchased from Laboratory Animal Centre of Dalian Medical University (Dalian, China; permit number: SCXK2013-0003). B16 cells (1 × 10^6^ cells in 200 μL PBS) were subcutaneously injected into the left flank of the above C57BL/6 mice [40]. After the melanoma could be touched, the C57BL/6 mice were randomly divided into four groups (five mice per group) and intraperitoneally injected with normal saline, 0.075 mg/kg, 0.15 mg/kg, as well as 0.3 mg/kg rLj-112 every day. Melanoma growth was calculated by the formula: (length × width^2^)/2. Thereafter, the length and width of the melanoma were measured with calipers. The above mice were sacrificed after 21 days and tumors were removed and measured. All animal procedures were conducted in accordance with the guide for the care and use of laboratory animals.

### 4.9. Statistical Analysis

Statistical analysis was performed with GraphPad Prism 5 software (GraphPad Software, La Jolla, CA, USA). Statistical significance was set as followed: *, *p* < 0.05; **, *p* < 0.01; ***, *p* < 0.001. Data were expressed as mean ± SD and analyzed using the Student’s *t*-test.

## 5. Conclusions

In conclusion, rLj-112, a mutant of rLj-RGD3 in which the three RGD motifs have been removed, also characterized by its high histidine content, was proved to inhibit the proliferation of B16 cells by inducing apoptosis in a caspase-dependent manner. Similar to rLj-RGD3, rLj-112 was also capable of suppressing the metastasis of B16 cells by disturbing the cytoskeleton arrangement. Furthermore, the internalized rLj-112 was also found to induce the internalization of EGFR and suppress the downstream signaling pathways to initiate the anti-tumor steps in B16 cells (Figure 9). In vivo studies, rLj-112 was shown to inhibit the growth, weight, and volume of the tumors in xenografted C57BL/6 mice without changing their body weights, suggested that rLj-112 might be safe and might have the potential to be used as a novel anti-tumor drug in clinical studies.

## Supplementary Materials

The following are available online at http://www.mdpi.com/1660-3397/17/2/75/s1, Figure S1: rLj-112 changed the protein levels of caspase 3, cleaved caspase 3, cleaved caspase 7, and cleaved PARP in B16 cells. Western blots (original images) showed the level of caspase 3, cleaved caspase 3, cleaved caspase 7, and cleaved PARP in B16 cells treated with PBS, 0.5, 1.5 and 2.5 μM rLj-112. GAPDH was used as a loading control, Figure S2: rLj-112 suppressed the activation of EGFR pathway in B16 cells. Western blots (original images) showed the levels of Akt, p-Akt, PI3K, p-PI3K, p-EGFR, p-p38 and p-ERK1/2 in B16 cells treated with PBS, 0.5, 1.5 and 2.5 μM rLj-112, respectively. GAPDH served as a loading control.
