# Supplementary material for: A Novel Mutant of rLj-RGD3 (rLj-112) Suppressed the Proliferation and Metastasis of B16 Cells through the EGFR Signaling Pathway
Source: Mar Drugs. 2019 Jan 23;17(2):75. doi: 10.3390/md17020075 (PMC6409697; doi:10.3390/md17020075)
Supplement: Supplementary file 1 [file marinedrugs-17-00075-s001.pdf]

Supplementary Materials:

A Novel Mutant of rLj-RGD3 (rLj-112) Suppressed the Proliferation and Metastasis of B16 Cells through the EGFR Signaling Pathway

Yuan-Yuan Zheng [1,†], Rong Xiao [1,†], Lu-Xin Zhang [1], Hui-Jie Yan [1], Ji-Hong Wang [1,*] and Li Lv [2,*]

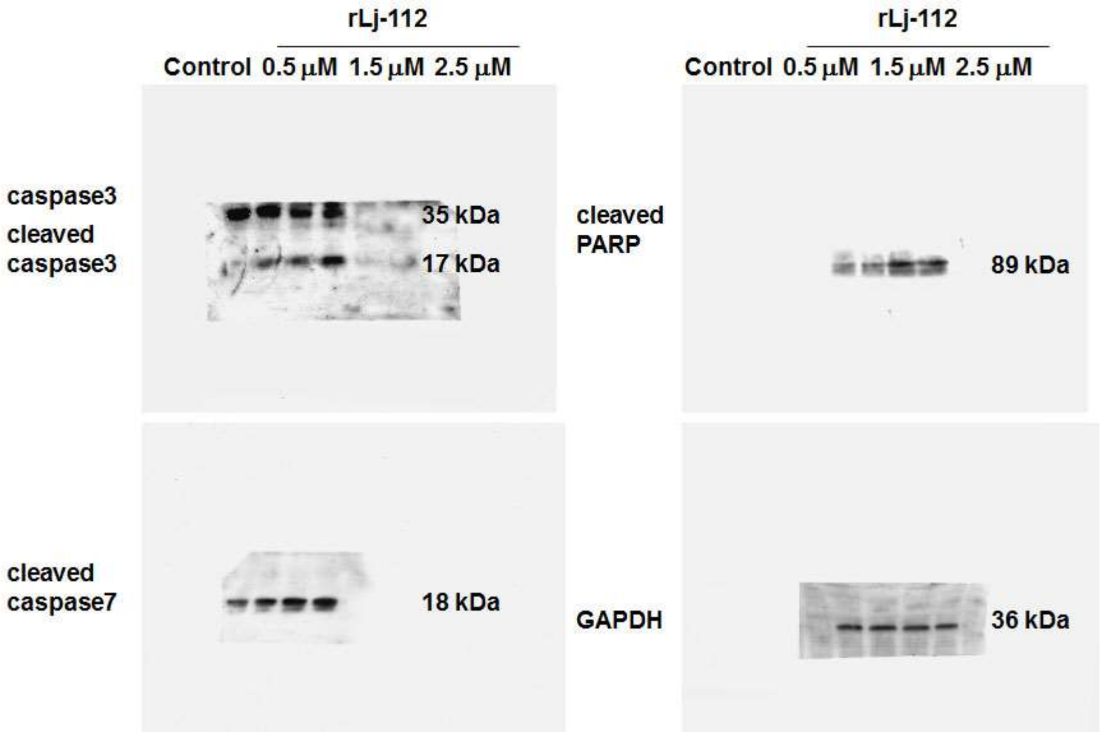

Figure S1. rLj-112 changed the protein levels of caspase 3, cleaved caspase 3, cleaved caspase 7, and cleaved PARP in B16 cells. Western blots (original images) showed the level of caspase 3, cleaved caspase 3, cleaved caspase 7, and cleaved PARP in B16 cells treated with 0, 0.5, 1.5 and 2.5 μM rLj-112. GAPDH was used as a loading control.

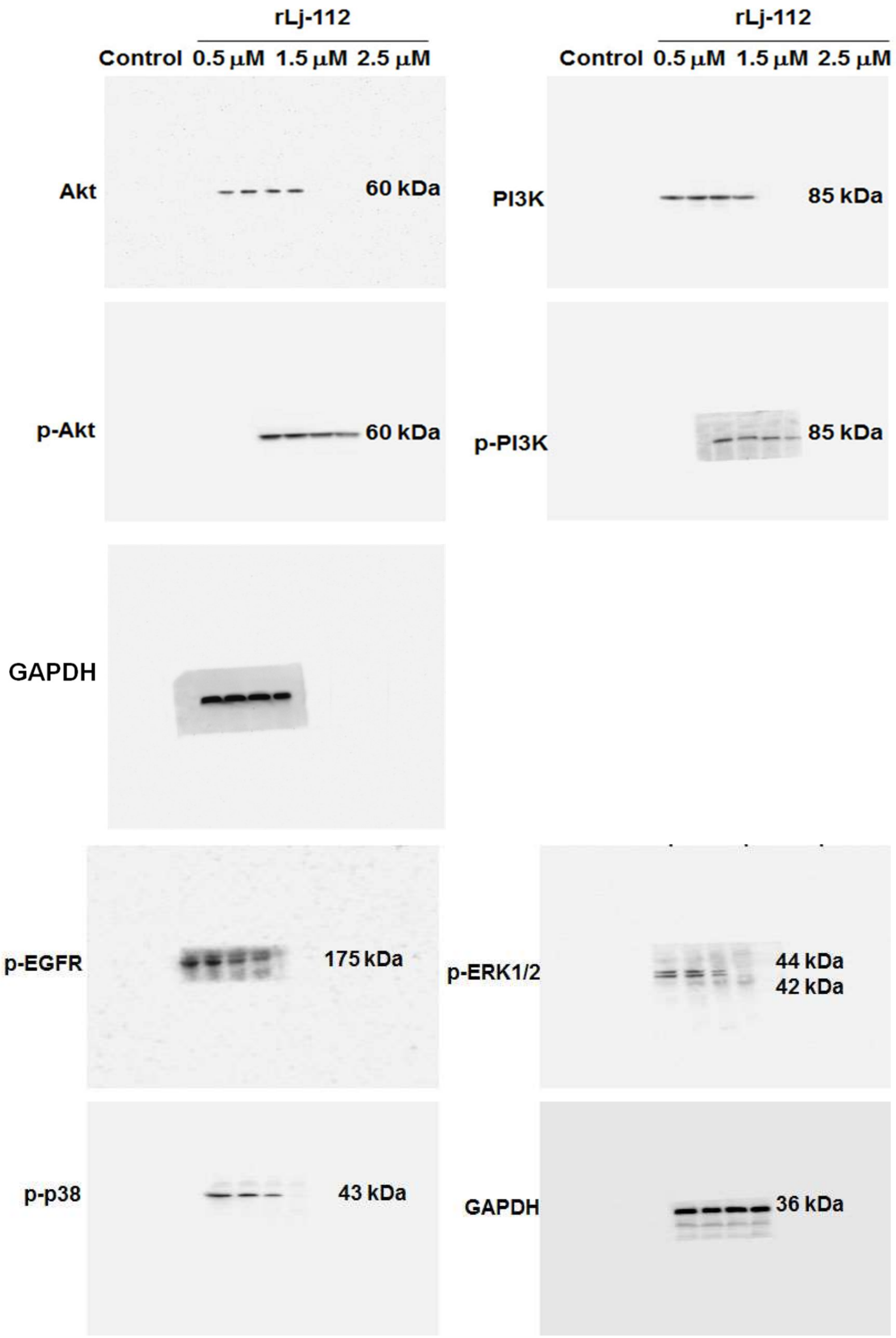

Figure S2. rLj-112 suppressed the activation of EGFR pathway in B16 cells. Western blots (original images) showed the levels of Akt, p-Akt, PI3K, p-PI3K, p-EGFR, p-p38 and p-ERK1/2 in B16 cells treated with 0, 0.5, 1.5 and 2.5 μM rLj-112, respectively. GAPDH served as a loading control.
